# Preclinical dosimetry: exploring the use of small animal phantoms

**DOI:** 10.1186/s13014-019-1343-8

**Published:** 2019-07-31

**Authors:** Emma R. Biglin, Gareth J. Price, Amy L. Chadwick, Adam H. Aitkenhead, Kaye J. Williams, Karen J. Kirkby

**Affiliations:** 1 Division of Cancer Sciences, University of Manchester, Manchester Cancer Research Centre, 3rd floor Proton Beam Therapy Centre, Oak Road, Manchester, M20 4BX UK; 2 The Christie NHS Foundation Trust, Manchester, UK; 3 Division of Pharmacy and Optometry, University of Manchester, Manchester, UK

**Keywords:** Radiotherapy, Tissue-equivalent, Dosimetry, Phantoms, 3D printing

## Abstract

Preclinical radiotherapy studies using small animals are an indispensable step in the pathway from in vitro experiments to clinical implementation. As radiotherapy techniques advance in the clinic, it is important that preclinical models evolve to keep in line with these developments. The use of orthotopic tumour sites, the development of tissue-equivalent mice phantoms and the recent introduction of image-guided small animal radiation research platforms has enabled similar precision treatments to be delivered in the laboratory.

These technological developments, however, are hindered by a lack of corresponding dosimetry standards and poor reporting of methodologies. Without robust and well documented preclinical radiotherapy quality assurance processes, it is not possible to ensure the accuracy and repeatability of dose measurements between laboratories. As a consequence current RT-based preclinical models are at risk of becoming irrelevant.

In this review we explore current standardization initiatives, focusing in particular on recent developments in small animal irradiation equipment, 3D printing technology to create customisable tissue-equivalent dosimetry phantoms and combining these phantoms with commonly used detectors.

## Background

Radiation studies using mice span decades, creating a large database of results. Translational research requires a preclinical in vivo model to facilitate the shift from in vitro results into clinical applications [1]. As radiotherapy (RT) clinical techniques evolve there is concern that data collected from mouse irradiation does not accurately represent the highly non-uniform focal or conformal dose distribution typically delivered to human patients [2]. Poor reporting of methodologies - affecting the reproducibility of experiments - undoubtedly contributes to the problem, but the central issue has been the difference between human and small animal irradiation techniques. Whereas human RT treatment machines have undergone huge technical development in recent decades and are now capable of delivering highly conformal dose distributions, many animal studies still utilise crude techniques targeting the whole body or using simple partial shielding [2, 3]. In an ideal scenario, mouse models would be used to map all aspects of human cancer treatment, multimodality combinations of surgery, chemotherapy, RT (using a range of doses and/or irradiation of a specific organ) and any new therapies as they develop. However, the lack of conformal irradiation units designed specifically for these mice models has hindered this goal [4].

Many studies have been initiated with the intent to find a method of animal radiation that reflects precise human treatment, due to the high potential animal models have of progressing research and improving RT (reviewed in [5]). Small animal irradiation was first proposed in the early 1970s. Early modalities include using cesium-137 or cobalt-60 sources, kilovoltage (kV) X-ray units and clinical linear accelerators [1–3]. The first example of a more clinically-familiar micro-irradiation unit was comprised of an iridium source, imaging system, motor controlled platform, and a collimator assembly with a computer to oversee the experiments [4]. Refinements in small animal RT techniques have led to higher precision treatment, image-guided RT, and dose escalation. However, the absence of dosimetry standards and poor reporting of dosimetry techniques in preclinical research is concerning as it limits the ability to compare and combine experimental cohorts between laboratories, and restricts reproducibility [5]. The causes of these issues are multifactorial and include a lack of awareness of the importance of rigorous radiation quality assurance amongst preclinical scientists leading to a paucity of dosimetric measurements, insufficient support from clinical physics and dosimetry colleagues, and inadequate equipment to undertake the task [6, 7].

As new technologies and approaches advance clinical RT techniques, their laboratory equivalents have been neglected [8]. Verhaegen et al. [3] hypothesise that the longer it takes for up to date preclinical RT to be developed the more likely it is that current radiobiological models become irrelevant. It is only recently that small animal irradiation units have begun to be developed to more closely mimic clinical equipment. It is now important that these advances are mirrored by the development of rigorous protocols and standardised equipment to modernise preclinical radiotherapy quality assurance. In clinical practice a series of standardised measurement phantoms and materials are commonly used, making it easy to compare and audit quality assurance (QA) techniques between centres. A similar approach would be valuable in the preclinical community. In this article we report current preclinical irradiation QA practice before reviewing the development of both small animal dosimetry phantoms, and the current state-of-the-art in small animal precision irradiation devices.

## The standardization of dosimetry

Dosimetry-related equipment and protocols in the clinical setting are well defined and regular QA and quality control is performed to ensure everything is working within defined tolerances [9]. The importance of the precision of dosimeters is highlighted in the requirement of regular calibration to a national standard:

1. A primary standard is nationally maintained at a dedicated dosimetry laboratory.
2. This provides a calibration factor for a mobile secondary standard requiring re-calibration every 3 years.
3. This secondary standard is used within a hospital to calibrate dosimetry equipment annually [9].

There is no legal requirement for this protocol to be followed at a preclinical level. In addition to the uncertainty introduced by not having properly calibrated equipment, uncertainty in dose can reach high levels if the following factors are not reported: beam energy, dose rate, temperature and pressure (when using detectors such as alanine pellets), fractionation regime, target volume and dosimeter depth [5]. Enforcing dosimetry standards in pre-clinical radiobiology will increase confidence in scientific results and encourage wider multicentre studies by improving comparability and reproducibility.

### Current methods of preclinical dose measurements

Mouse models are considered ideal investigative tools for research as they offer established genetic strains and produce efficient results translatable to humans [10]. However, their heterogeneous density and intricate anatomy make both simulating and measuring delivered dose difficult [11]. A way to minimise this uncertainty could be identifying the most contrasting densities - bone and lung - and measure the dose delivered to these targets [12]. Another major source of uncertainty is the scattering processes, even when in reference to established protocols [13]. The AAPM TG-61 protocol is the reference outlining dose rate for energies up to 300 kV. This protocol is based on in–air measurements of the entrance surface dose of a water phantom, with tabulated backscatter correction factors. However, these scattering conditions are very different to those during preclinical irradiations with small heterogeneous targets with irregular surface geometry. Noblet et al. [13] investigated this difference and found that the lack of backscatter seen when using small, irregularly shaped targets (compared to a water phantom) causes a more rapid dose rate decrease. Without accounting for appropriate scatter conditions the measured dose will be an underestimation of that delivered.

#### Phantoms

Phantoms are used in radiation dosimetry (clinically and preclinically) to investigate and measure the effects of dose on an organ or tissue. They can be composed of water or more complex materials to closely resemble components of a body, in defined shapes and sizes [14]. Inter-centre dosimetry audits are periodically undertaken in the clinical setting and, less commonly, at preclinical facilities, to assess accuracy in delivered dose. Phantoms containing dosimeters are distributed to participating centres with explicit experimental protocols and the resulting measurements compared [5, 7]. Pedersen et al. [7] sent 6 acrylic phantoms with space for 3 thermoluminescent detectors (TLDs) to 12 radiobiology institutions. Each institution was instructed to deliver 1Gy to 3 of the phantoms and 4Gy to the others. Taking accidental exposure into account, the results showed a substantial average difference between the delivered and intended dose, ranging from 0.9 to 42%. To get an accurate representation of the irradiation procedures at each institution limited instructions were provided with participants asked to follow their own irradiation protocol [7]. Although this reduced the influence of bias, it is unclear how comparable the different centres’ irradiation protocols were to the conditions under which the reference TLD irradiation procedure was completed. Further work might consider accounting for different baseline calibrations and could replace the cylindrical phantoms with a heterogeneous density phantom to show a more accurate demonstration of in vivo radiation dosimetry.

#### Detectors

Detectors are commonly used in conjunction with a phantom for dosimetry measurements. Dosimeter function depends on properties such as linearity (the relationship between the dosimeter reading and dosimetric quantity), dose rate, energy dependence (the effect of different energies on the measurements), spatial resolution (the clarity of the dose map) and, in particle therapy, the energy transferred per unit length of the track – linear energy transfer [15]. A number of detectors have been well established in this field, summarised in Table 1.

**Table 1:** Summary of the detectors currently available [5, 16–19]

Detector	Specifications	Advantages	Disadvantages
Ionisation chambers	• Commissioning • Dose calibration • QA • Uncertainty: < 5% • Dose: up to 1000Gy	• High precision and accuracy • Various models, including waterproof models • No effect from dose rate • Instant readout	• Requires high voltage • Size • Elaborate care
Radio-graphic Film	• Imaging • Dosimetry • Phantom compatible • Uncertainty: < 5% • Dose: 0.1-5Gy	• Great spatial resolution • 2D dose distribution • No effect from dose rate • Various film types • Useful for assessing field size, flatness and symmetry	• Complex processing • Film type/batch variation • Dose calibration required • Affected by energy • Light sensitive • Not reusable
Radio-chromic Film	• Imaging • 2D Dosimetry • Phantom compatible • Uncertainty: < 5% • Dose: 0.1-200Gy	• Self-processing • Tissue-equivalent • No effect from dose rate/energy • Great spatial resolution • Useful for radiation field size, flatness and symmetry	• Results vary between film types and batches • Dose calibration required • Not reusable • Requires stabilisation period after irradiation
TLDs	• In vivo dosimetry • Phantom compatible • Audit purposes • Uncertainty: < 5% • Dose:<200Gy	• Small size • Multiple point readings • Various models available • Reusable	• Laborious calibration • Delayed results • Signal erased during readout • Results vary between batch • Light sensitive
OSLDs	• In vivo dosimetry • Phantom compatible • Audit purposes • Uncertainty: < 4% • Dose: <10Gy	• Moderate size • Multiple point readings • Quick readout • No effect from dose rate	• Light sensitive • Limited availability • Not suitable for calibration • Energy dependent
Silicon Diodes	• In vivo dosimetry • Detector arrays • Dosimetry • Uncertainty: < 3% • Dose: <10Gy	• Moderate size • Instant readout • Good sensitivity • No external voltage • Small field dosimetry	• Requires connecting cables • Temperature sensitive • Unsuitable for calibration • Unsuitable at higher doses
MOSFETs	• In vivo dosimetry • Small field dosimetry • Detector arrays • Uncertainty: < 5% • Dose: <10Gy	• Small size (0.2 × 0.2 mm) • Multiple point readings • quick readout • Good sensitivity	• Calibration required • Energy, temperature and directional dependent • Unsuitable for calibration
Diamond Detectors	• In vivo and small field dosimetry • Uncertainty: < 3% • Dose: <10Gy	• Small size • Tissue-equivalent • High sensitivity • Resistant to radiation	• External equipment required • Requires pre-irradiation • Results vary among detectors • Unsuitable for calibration
Alanine – Electron para-magnetic resonance	• In vivo dosimetry • Phantom compatible • Audit purposes • Uncertainty: < 4% • Dose: 10-150000Gy	• Tissue-equivalent • Readout process does not diminish signal	• Readout requires specific equipment
Gel dosimetry detectors	• 3D dosimetry • Audit purposes • Uncertainty: 5–10% • Dose: <10Gy	• Tissue-equivalent • Both phantom and detector • 3D dose distribution	• Elaborate preparation • Continued processing • Difficult reproducibility • Unsuitable for calibration

Key: *QA* quality assurance, *TLDs* thermoluminescent detectors, *OSLDs* optically-stimulated luminescent detectors, *MOSFETs* metal oxide semiconductor field effect transistors

## Creating small animal dosimetry phantoms

The earliest examples of “mouse” phantoms included hollow cylinders containing liquid, mathematical representations based upon measuring the size and mass of a mouse, voxel-based approaches and cuboids with integrated detectors [11, 20–22]. Technological advances have allowed the current generation of phantoms to be developed with varying shapes or densities more recognisable as a small animal, and recent developments are incorporating more heterogeneous densities [10, 11, 23]. Welch et al. [11] demonstrated the first construction of a phantom, based on cone beam CT (CBCT) data, with both the internal and external characteristics of a mouse. Individual slices were constructed of material mimicking soft tissues in both density and X-ray attenuation properties. Appropriate holes were then milled in these slices and filled with bone- (epoxy resin) or lung-equivalent material (urethane-based material with polystyrene microbeads) [10]. The materials used to create this phantom are only available at 2 mm thickness, creating an uneven ‘stepped’ surface, limiting the resolution of the phantom and restricting the detail of smaller regions of heterogeneity. The milling process to create areas to be filled with different materials is also laborious, restricting production to institutions and companies who have the appropriate machinery, and if performed manually may impact reproducibility.

### 3D printing

In recent years 3D printing has been widely utilised in the manufacturing of radiotherapy phantoms. It is cost effective, efficient, capable of submillimetre accuracy, and can make use of a wide variety of materials [24]. Fused deposition modelling (FDM) and stereolithography are the most commonly used techniques for 3D printing. FDM creates the model by melting a thermoplastic, most commonly acrylonitrile butadiene styrene and polylactic acid, and depositing it in layers. Stereolithography utilises photopolymer resin formed into layers using an ultraviolet laser [24]. FDM is the cheaper option for 3D printing but is less accurate than stereolithography, which may cause problems when creating small or irregular voids, or when printing intricate anatomy such as a mouse spine and ribs. Furthermore, the FDM process can unintentionally incorporate small air gaps between depositions potentially affecting reproducibility. Another phantom construction method that has been used is to 3D print the outside shell and important internal structures, such as the skeleton, and then fill the void with a tissue-equivalent liquid [23]. With care this could reduce the risks of creating air gaps in the material, but may require non-anatomical support structures to correctly position the internal structures within the body surface shell, as well as requiring that the printed shell must be completely watertight.

### Incorporation of dosimeters

A phantom constructed of slices allows the incorporation of interchangeable slices with an integrated detector, or can incorporate Gafchromic film between layers [10, 11]. Another way of incorporating space for dosimeters is to modify the model before 3D printing by using Boolean operations to create holes, print the model in segments to allow film to be sandwiched in different orientations, or print a hollow design to fill with a liquid detector [25, 26]. An advantage of 3D printing models is being able to design the hole to precisely fit the specific detector thereby reducing the geometric uncertainty and the risks of surrounding air gaps [27].

### Tissue-equivalent phantoms

Categorising a material as “tissue-equivalent” suggests the composition has identical radiation characteristics and physical properties, when exposed to a defined energy range, as the tissue it represents. Developing phantoms that mimic both the material properties and anatomical shape of real mice permits the measurement of doses that account for the effects of both the beam attenuation and X-ray interaction processes that would occur during real experiments [6, 28]. When considering the materials being used for tissue equivalence it is also important to consider the conditions of the experiment to determine what properties to mimic [5].

To create tissue-equivalent materials (TEMs) it is common to combine a plastic, for stability, with an additional substance to produce the desired density and attenuation. For example, to create a soft tissue-equivalent material Winslow et al. [29] mixed two parts urethane with one part calcium carbonate whilst a bone-equivalent material was created using an epoxy resin blend with silicon dioxide and calcium carbonate. Another way of adapting density to suit a specific tissue type is the inclusion of particles in the mixture. This is commonly seen when creating lung-equivalent areas, for example, distributing polystyrene microbeads within a TEM to represent different amplitudes in the breathing cycle [11, 29]. The above material recipes were developed for diagnostic imaging (X-ray energy 80-120kVp) and match the density, X-ray attenuation and energy absorption of soft tissue and bone well within this range. However, typical radiobiological irradiations use higher X-ray potentials (up to 300 kVp) [3]. The use of 3D printing technology permits further modification of material properties by varying the way in which the printed materials are deposited - the modification of layer formation and infill density permits the creation of highly accurate and customisable tissue-equivalent models [24]. Perks et al. [26] utilised this method to simulate lungs by purposely incorporating air gaps in the grid-structured print creating a model of 1/3 density. The next step could be creating multiple models with different grid structures to mimic different stages in the breathing cycle.

A state of the art dosimetry phantom would include all of the aforementioned properties. 3D printing using tissue-equivalent materials (for energies in the 10-300 keV range) creates a bespoke phantom suitable for imaging and radiation delivery QA. It is easily reproducible, can be combined with a range of detectors and is cost effective, allowing laboratories to manufacture and modify their own phantoms. Increased use of such phantoms could be encouraged by distributing a standard phantom with QA equipment or by offering an open source CAD file of the phantom. Reaching a consensus as a community and adopting a common phantom design and QA approach would be a big step towards improved reporting and experimental repeatability.

## Small animal irradiation units

**Fig. 1:**
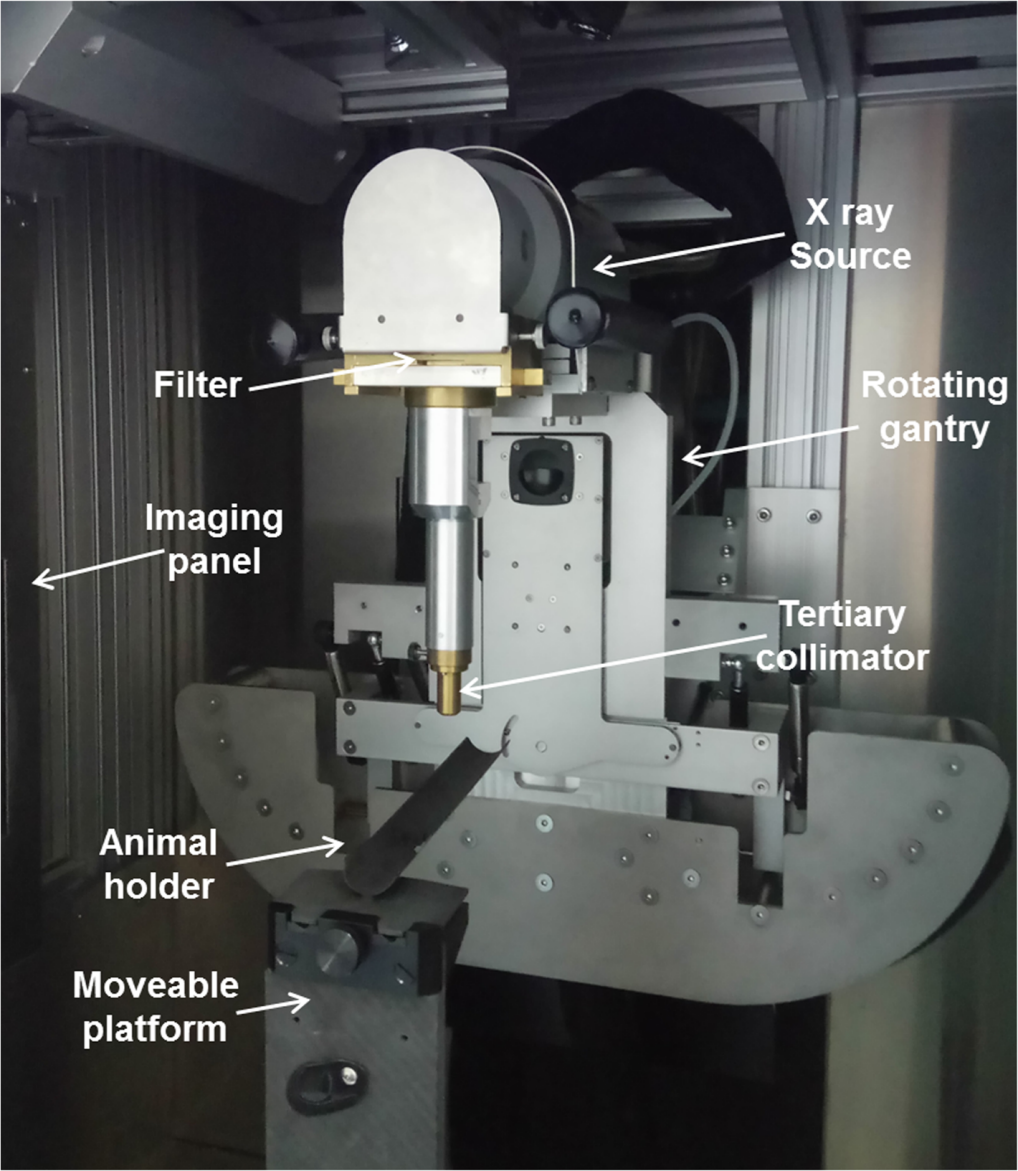
The small animal radiation research platform (Xstrahl, Ltd). With the aim of reflecting human radiotherapy the small animal radiation research platform has a rotating gantry, image guidance and moveable platform, all controlled through an accompanying treatment planning system

Since 2008 several small animal radiation systems have been developed (reviewed in [3], Table 2). Recent developments include increasing beam delivery to submillimetre accuracy, improving the dose delivered to within 5% of planned dose and increasing the number of treatment positions from the four cardinal angles. It is essential that the radiation techniques utilised by these machines mirror those used in radiotherapy on humans (Fig. 1), including the ability to target small areas seen in stereotactic cranial irradiation and dose painting across the treatment field using a variable collimator [30, 31]. Small animal irradiation, compared to clinical machines, depends on a design that requires adaptation to: beam quality, radiation dose and dose rate, irradiation time, field size and source to surface distance (SSD) [5, 6].

**Table 2:** Characteristics of the developed small animal irradiators [3]

Model	Source	Imaging	Positioning	Additional
SARRP (Xstrahl Ltd)	X-ray, 5–225 keV	Amorphous Si flat panel detector for dual imaging system (CT) and planar X-ray	Robotically-controlled stage, 35 cm SSD, 4 degrees of freedom. Allows continuous radiation delivery either from rotating gantry or platform.	2 collimation systems: 1 for precision with smaller, conformal inserts, another for higher throughput with larger square field sizes.
X-Rad 225Cx (Precision X-Ray Inc)	X-ray, 5–225 keV	Amorphous Si flat panel for single image or cone beam CT.	3D computer controlled stage with automated corrections	Selection of beam collimators providing 0.2 mm accuracy
Washington University	Iridium 192 (brachytherapy)	N/A (fiducial markers)	Computer controlled stage, 4 gantry angles	Tungsten collimators 5-15 mm
Stanford University	X-ray, 70–120 keV (microCT scanner)	Designed for small animal imaging so 0.1 mm spatial resolution	Arc or fixed field	Brass iris collimators (0.1-6 cm field sizes
University of Texas Southwestern	X-ray 5–320 keV	Fixed panel	3D precision stage, cylinder for immobilisation	Cylindrical collimators 1-10 mm

Key: *SARRP* small animal radiation research platform, *keV* kiloelectron volts, *CT* computed tomography, *SSD* source to surface distance

Small animal RT requires precise targeting, high resolution imaging capability and appropriate dose verification technology [3]. Equipment should include an X-ray tube (kVp: 10-320 kV), collimating device, generator and controls to set the beam energy, tube current and time. With small animal irradiation megavoltage beams may be too high an energy which would lead to insufficient surface dose, increased lateral scatter and hotspots at depth [6].

### Facilities

To achieve appropriate field sizes for small animals these machines should aim to achieve submillimetre field sizes, which introduces strict tolerances on the mechanical accuracy of the machine. For example the microRT device developed by Kiehl et al. [32] can produce conformal beams with an accuracy of ±0.2 mm. Once submillimetre field sizes are routinely implemented it may be necessary to introduce higher resolution detectors, such as diamond detectors, into the QA procedures. The ability to accurately target the tumour, whilst sparing normal tissue, is the main goal of radiotherapy. One way to achieve optimal conformation is the use of a multi-leaf collimator that can create intricate shapes to best fit the tumour area. Until recently, small animal irradiators achieve this by the use of multiple fixed-shape collimators that are manually changed during the treatment. Cho et al. [31] developed a variable rectangular collimator suitable for use on the SARRP creating a dose painting effect using a series of rectangular geometries. The main limiting factor when using submillimetre field sizes is the reduction of dose rate meaning a suggested minimum of 20 cGy min^− 1^ may not always be achievable. For example, at a depth of 6.15 cm, using a 0.5 mm diameter field size peak dose rates of 18.7 cGy/min and 10.9 cGy/min were achieved by Tryggestad et al. [33] at 34 cm and 38 cm SSD respectively, but all measurements at shallower depths achieved dose rates of above 20 cGy/min. Also reflecting current clinical practice, it should be possible to target the model from a variety of angles, or as a continuous arc treatment.

#### Target platform

For repeatable experiments, fractionated schedules and efficient use, small animal units have a motorised positioning stage that may be equipped differently for specific purposes using either individual restraining devices or removable carbon fibre animal beds [1]. These platforms can move in the X, Y and Z directions and rotate 360° [34]. An adapted couch with acrylic dividers can be used to facilitate multiple animals/phantoms, increasing throughput, improving immobilisation and facilitating positioning for thoracic, abdominal and brain irradiation [25]. As with human RT, immobilisation devices have been developed to allow better targeted irradiation for more focused treatment such as stereotactic cranial irradiation [30]. McCarroll et al. [35] created a 3D printed immobilisation device, specifically based on the CT scan of a mouse to reduce animal motion during irradiation and allow for accurate and reproducible positioning. However, this extension of the moveable platform must be rigidly attached to avoid the introduction of additional motion uncertainties when moving the platform. The use of immobilisation devices will also likely increase treatment time which is something that must be considered both from experimental throughput and animal welfare points of view, particularly when animals are anesthetised.

#### Imaging and tissue segmentation

Treatment plans have been numerically simulated on patients’ CT scans for decades and image guidance is the standard of care in the clinic. Modern small animal irradiators now mimic this workflow [3] but the process is more complicated as the calculation of dose requires more accurate definition of the elemental composition of tissues due to the prominence of the photo-electric effect at the kilo-voltage energies used in these platforms [36]. Comparing to clinical energies, Verhaegen et al. [37] suggests that at lower energies (220 kV) differences of dose measurement could reach 40% if tissue segmentation (and hence material property assignment) is inaccurate but at 6 or 15MV the same misalignment would lead to < 10%.

Schneider et al. [38] originally proposed the method by which to derive the elemental composition of a material from its CT data. It uses the Jackson and Hawkes equation to relate CT number, physical density and atomic number from the CT images of known materials. Noblet et al. [36] proposed using this method of assigning tissue properties as a means to calculate absorbed dose for small animal radiotherapy. They measured the relationship between CBCT number and the product of material density and elemental composition for a set of known materials. These data were then used to assign the correct properties in each voxel of small animal CBCT planning images. The authors validated their method by simulating the dose transmitted through a mouse with measurement and conclude that this method improves on bulk density overrides [36]. However, even using this approach the error remains higher (~ 4%) than the clinically accepted 2% tolerance highlighting the fundamental challenges of emulating clinical workflows in the preclinical environment and the importance of robust quality assurance.

For image guidance, micro-CT devices with smaller apertures and smaller X-ray tubes are available, working in the same way as standard CT scanners. Most small animal irradiators provide CBCT via a rotating turntable, a fixed source and amorphous Si flat-panel detector, whereby the mouse is rotated to create the desired image [3, 6]. Some models may have a second imaging system to acquire projection images to evaluate the movement of the stage and feasibility of the rotation for the CBCT or to confirm detector positioning [25, 34].

#### Treatment planning system (TPS)

As with clinical RT treatment plans defining beam directions, collimation, and dose are developed on CT images. The planning images are typically acquired using the irradiator’s on-board CBCT system which may be used to distinguish internal structures or identify fiducial markers placed in tumours to allow precise targeting [3]. CBCT imaging has intrinsically poorer image quality than diagnostic images. For this reason alternative modalities such as bioluminescence imaging, magnetic resonance imaging and standard CT can also be utilised in the treatment planning process [30].

However, there are further developments still required. TPS commissioning is still problematic and rigorous validation using anatomically realistic phantoms should be undertaken as it is in the clinic. There is still more research needed on photon scatter at kV energies and when using narrow beams. Furthermore, some TPSs still rely on bulk density overrides from tissue segmentations that both increases dependence on their accuracy and masks the heterogeneity effects that will affect the actual dose delivered. [39]. Monte Carlo codes (FLUKA or GEANT4) are being incorporated into TPSs to try and improve upon dose modelling quality [40].

### Quality assurance

As discussed in our introduction, lack of quality assurance of irradiation facilities in radiobiology labs risks undermining much of the subject’s foundation. One of the core principles of the scientific method is open reporting and repeatability of experiments. Without accurate knowledge of the doses delivered in experiments this principle is put at risk. The unique design of scaled down components in small animal units require specialised tools and methods for robust QA [41]. Most common daily output measurements of the SARRP are completed with a solid water phantom and an ionisation chamber. However, unless multiple points are measured this does not provide information about the distribution of the beam. One phantom design currently recommended for the QA of the SARRP is the Mousefet phantom as designed by Ngwa et al. [41], which is particularly useful as it can be used for the verification of both the imaging and irradiation apparatus as it has an arrangement of MOSFET detectors within the 3D phantom. However this is very reliant on accurate positioning when using small beams as it is easy to place a detector at the field edge by accident. Phantoms can be designed to perform daily, monthly and annual QA [42]. Examples include the ball bearing phantom to ensure accurate mechanical alignment, a quick procedure undertaken regularly, and the exhaustive beam quality tests using solid water slabs (60 mm × 60 mm x 5mm^3^) described below, used for commissioning and annual system checks of the SARRP platform. Whilst such approaches can be used to assure beam quality and systems’ geometric accuracy, they cannot assure the quality of the delivered prescription – such assessments require end-to-end testing, often using anatomically realistic phantoms. Undertaking such testing is deterred by a lack of dosimetric expertise or restricted access to appropriate calibrated equipment. This problem can be partially addressed by the provision of equipment designed for the purpose, but will also require a greater investment in acquiring the necessary skills – either through appropriate training of laboratory staff, or through collaboration with medical physics departments where the skill base already exists.

### Commissioning the small animal radiation research platform

**Fig. 2:**
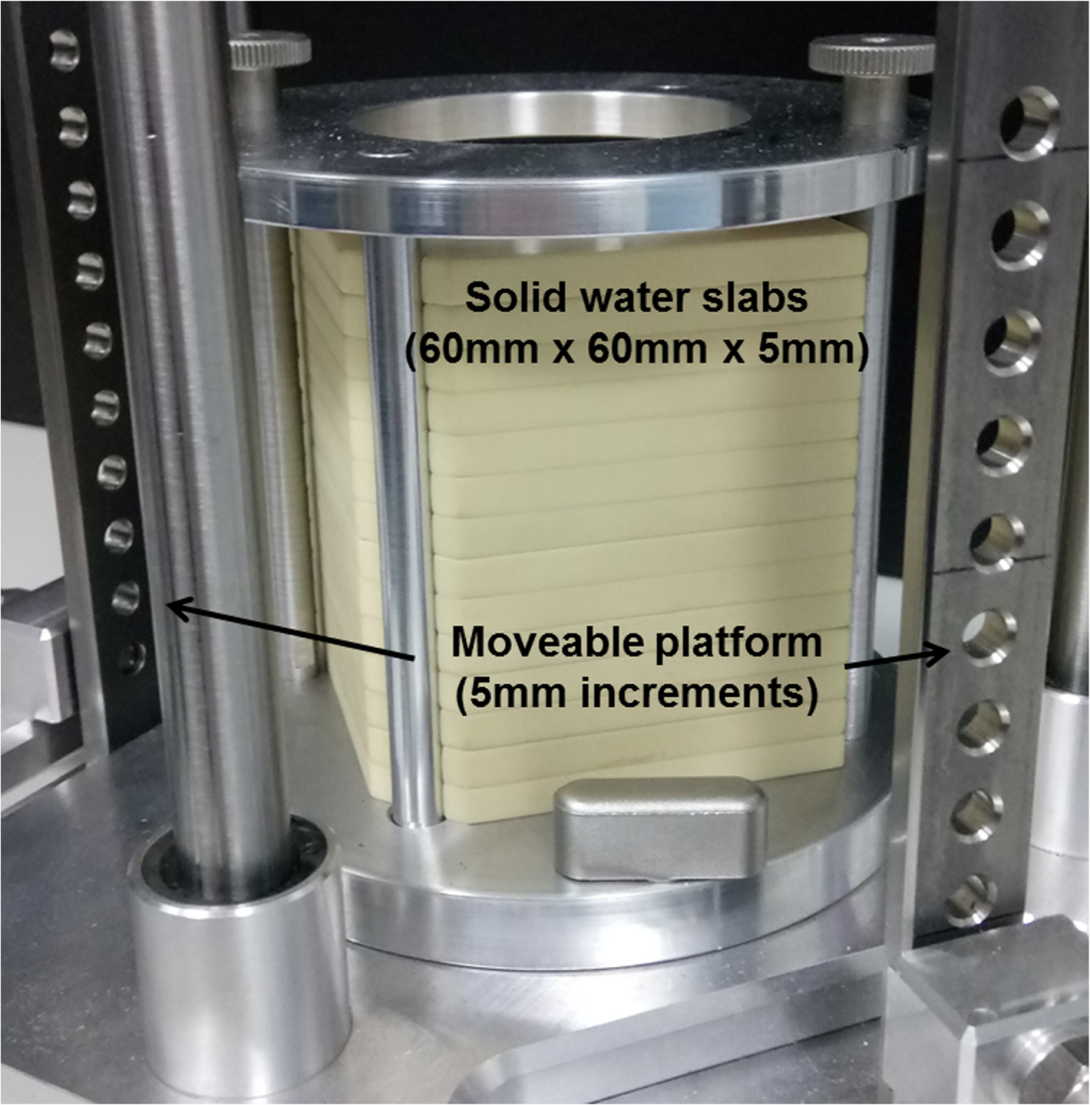
The small animal radiation research platform (Xstrahl, Ltd) commissioning jig. Solid water slabs 60 cm × 60 cm × 5 cm are arranged in a stack to create a phantom appropriate to incorporate layers of film at defined intervals to take measurements of dose to create a depth dose profile

The commissioning of an irradiator should allow the characterisation of dosimetric properties such that the dose delivered is accurate within 5% [43]. For the commissioning of the SARRP platform, Gafchromic EBT film, calibrated relative to an ion chamber at different exposures, is sandwiched between fifteen layers of solid water 5 mm thick at specified intervals (Fig. 2) [33]. The jig holding the solid water slabs in place has the ability to move along an axis to allow variable SSD measurements, between 32 and 38 cm (5 mm increments). This set up allows an accurate assessment of a percentage depth dose in addition to the flatness, symmetry and penumbra of the beam profile. Each brass collimator must be individually assessed using this set-up. [33]. As discussed above, whilst this process ensures that the beam quality is within tolerance, it does not test for the myriad of other errors that can occur in the experimental workflow. It is vital that not only is each step quality assured in its own right, but that the whole process is also tested end-to-end.

## Conclusions

We have highlighted the potentially serious problems that the lack of rigorous quality assurance in preclinical radiation research can, and possibly has, caused. Not only is scientific quality at risk, we are ethically obliged to ensure that the data from each animal used in scientific research is fit for purpose and contributes to progress. Initiatives to address some of these problems have already been started, but there is still work to be done.

We have discussed developments in the QA of individual steps of the workflow in small animal irradiators. However, much of this work takes place at individual institutes with limited collaboration. There is a need for the whole international community to come to a consensus and adopt standardised QA protocols and equipment. By combining biologically-relevant phantoms with the latest developments in detector technology it will be possible to conduct rigorous end-to-end tests from initial imaging and plan development, through image guidance, to treatment delivery. One of the first steps on the path to standardization is to better understand the problem. We propose building on the work of Pedersen et al. [7] by undertaking audits using state-of-the-art phantom technology referenced to a national standard.

Developing rigorous QA protocols will drive quality, reducing dosimetric uncertainties, and, importantly, ensuring each animal used in experiment is contributing to scientific progress. Accurate treatment planning, precision targeting and arc irradiations will further close the gap between the techniques seen in the clinical and preclinical settings. The development of new therapies is reliant on preclinical experiments. However, only a third of animal research continues on to human randomised trials [44]. If experiments do not reflect the clinical reality there is risk that results will not be translatable. By ensuring a close match between the preclinical and clinical radiation treatments this risk will be reduced [44]. Increased used of precision irradiators, coupled with a concerted effort to adopt standardised QA procedures will be a large step in this direction.

## Data Availability

Not applicable.

## Abbreviations

CBCT: Cone beam computed tomography
FDM: Fused deposition modelling
keV: kiloelectron volts
KV: Kilovoltage
MOSFETs: Metal oxide semiconductor field effect transistors
OSLD: Optically-stimulated luminescent detectors
QA: Quality assurance
RT: Radiotherapy
SARRP: Small animal radiation research platform
SSD: Source to surface distance
TEM: Tissue-equivalent material
TLD: Thermoluminescent detector
TPS: Treatment planning system
